# The House of Steinway

**Published:** 1875-02

**Authors:** 


### THE HOUSE OF STEINWAY.

In our last number we gave a list of articles used by the great House of Steinway in the construction of one of their famous pianos, describing at the close the timber and iron frame-work upon which the delicate instrument is built. The construction of the case, the resonant box which encloses the working-parts—is a work of extensive difficulty, on account of the size and slightness of the structure, and the adverse influences to which it is exposed in our trying climate. To give lightness and strength and resonant qualities, the solid, well seasoned boards are covered with a double veneer designed to counteract all the tendencies to warp; and the surface is laboriously polished. It takes the Steinways three months to varnish and polish the case of a piano. Fully a thousand cases in process of finishing may be seen in this factory at any time. When the surface of the wood has been made as smooth as possible, the first coat of varnish is applied, and this requires eight days to harden. Then all the varnish is scraped off, until the surface of the veneer is laid bare. Of course considerable varnish has soaked into the grain of the wood. Another coat is laid on and scraped off after eight days. Then the pores of the wood are deemed full, and on this substructure five or six coats of colorless varnish are laid in about six weeks. Each coat must become as hard as possible before another is applied. Thus this thin, transparent film is satisfactorily formed and hardened, protecting the wood and in no way disguising its delicate beauty, but rather heightening its tint and tracery. Then a prolonged rubbing-process, terminating with the naked hand of the workman, and the finish is complete. The object of all this is not merely to produce a splendid and enduring gloss, but to make the case stand for generations in a room which is heated by a furnace to eighty or ninety degrees by day, and in which water will freeze at night. This finishing process is a very expensive one, not only on account of the high cost of the materials used, but especially because of the immense labor involved. During the war the limpid varnish used on pianos cost as much as the best champagne.

But the labor is the chief item of expense. The average wages of the laborers—varying in number from six hundred to a thousand—employed by the Messrs. Steinway, is twenty-six dollars a week. This force, aided by one hundred and two labor-saving machines, driven by steam-power equivalent to two hundred horses, produces a piano in one hour. A man with the ordinary tools can make a piano in four months, but it could not possibly be as good a one as those produced in a large establishment. The small makers, who manufacture from one to five instruments a week, are in the habit of buying the various parts from persons who make only parts. It is a business to make the hammers of a piano—the bits of felt-clad wood which strike the strings when the keys are depressed; it is another business to make the "action;" another to make the keys of ivory and ebony; another the legs; another the cases; another the pedals. The manufacture of the hardware used in a piano is a very important branch, and it is a separate business to sell it. With most makers the labor is considered only half accomplished with the completion of the instrument. With the House of Steinway, the labor is practically completed when the instrument is done, so great is the demand for the products of their establishment.

